# Structural brain characteristics in treatment-resistant depression: review of magnetic resonance imaging studies

**DOI:** 10.1192/bjo.2019.58

**Published:** 2019-09-02

**Authors:** Margit Philomène C. Klok, Philip. F. van Eijndhoven, Miklos Argyelan, Aart H. Schene, Indira Tendolkar

**Affiliations:** Psychiatrist, Department of Psychiatry, Radboud University Medical Center, the Netherlands; Psychiatrist, Department of Psychiatry, Radboud University Medical Center; and Donders Institute for Brain Cognition and Behavior, Centre for Cognitive Neuroimaging, the Netherlands; Psychiatrist, Center for Psychiatric Neuroscience, The Feinstein Institute for Medical Research; and Division of Psychiatry Research, Zucker Hillside Hospital, Northwell Health, USA; Professor of Psychiatry, Department of Psychiatry, Radboud University Medical Center; and Donders Institute for Brain Cognition and Behavior, Centre for Cognitive Neuroimaging, the Netherlands; Professor of Psychiatry, Department of Psychiatry, Radboud University Medical Center; Donders Institute for Brain Cognition and Behavior, Centre for Cognitive Neuroimaging, the Netherlands; and LVR-Hospital Essen, Department for Psychiatry and Psychotherapy, Faculty of Medicine, University of Duisburg-Essen, Germany

**Keywords:** Structural characteristics, neuro-imaging, therapy resistant depression

## Abstract

**Background:**

Major depressive disorder (MDD) has been related to structural brain characteristics that are correlated with the severity of disease. However, the correlation of these structural changes is less well clarified in treatment-resistant depression (TRD).

**Aims:**

To summarise the existing literature on structural brain characteristics in TRD to create an overview of known abnormalities of the brain in patients with MDD, to form hypotheses about the absence or existence of a common pathophysiology of MDD and TRD.

**Method:**

A systematic search of PubMed and the Cochrane Library for studies published between 1998 and August of 2016 investigating structural brain changes in patients with TRD compared with healthy controls or patients with MDD.

**Results:**

Fourteen articles are included in this review. Lower grey matter volume (GMV) in the anterior cingulate cortex, right cerebellum, caudate nucleus, superior/medial frontal gyrus and hippocampus does not seem to differentiate TRD from milder forms of MDD. However, lower GMV in the putamen, inferior frontal gyrus, precentral gyrus, angular- and post-central gyri together with specific mainly parietal white matter tract changes seem to be more specific structural characteristics of TRD.

**Conclusions:**

The currently available data on structural brain changes in patients with TRD compared with milder forms of MDD and healthy controls cannot sufficiently distinguish between a ‘shared continuum hypothesis’ and a ‘different entity hypothesis’. Our review clearly suggests that although there is some overlap in affected brain regions between milder forms of MDD and TRD, TRD also comes with specific alterations in mainly the putamen and parietal white matter tracts.

**Declaration of interest:**

None.

Major depressive disorder (MDD) is the most common psychiatric disorder with a lifetime prevalence estimate of 9.4%.^1^ Although two-thirds of patients with MDD recover with current treatment approaches, the condition in one-third has a treatment-refractory course. When treatment does not result in remission, it is an unsuccessful trial, whether because of a lack of efficacy (i.e. lack of remission) or intolerable side-effects. The degree of treatment resistance is gauged by the number of treatment steps needed to achieve an adequate benefit.^2^ Treatment-resistant depression (TRD) is associated with prolonged, costly in-patient periods of treatment.^3^ The extent to which individuals with TRD versus treatment-responsive MDD differ in terms of aetiology or pathophysiology remains mostly obscure. Early-life stress is hypothesised to increase treatment resistance^4^ and individuals with TRD may exhibit differences in brain circuit function^5^ but nevertheless the underlying mechanisms of therapy resistance are not yet know;^6^ these demand new insights regarding the underlying pathophysiology and for treatment approaches. Advances in neuroimaging techniques may allow us to identify structural differences in grey matter in MDD and relate it to the severity of disease.

## Structural characteristics in MDD

Koolschijn and colleagues^7^ were the first to conduct a meta-analysis on regions of interest (ROIs) extracted from six studies including a total of 2418 patients with MDD and 1974 healthy individuals. Patients showed significant volume reductions in frontal regions, in particular in the anterior cingulate cortex (ACC) and the prefrontal cortex, most pronounced in the orbitofrontal cortex. The hippocampus, the putamen and caudate nucleus showed moderate volume reductions. The authors concluded that MDD is associated with structural brain abnormalities, particularly in those brain areas involved in emotion processing and stress regulation. To account for potential confounding because of different methods Bora and colleagues^8^ conducted the first coordinate-based meta-analysis of voxel-based morphometry (VBM) studies in MDD. They included 23 studies with a total of 986 patients with MDD and 937 healthy controls. The authors could confirm a significant reduction in GMV in the ACC and in the dorsolateral and dorsomedial prefrontal cortex and conclude that grey matter reduction in rostral ACC is the most consistent finding in VBM studies of MDD. Arnone *et al*^9^ used a novel voxel-based technique based on the statistical parametric maps from individual magnetic resonance imaging (MRI) studies, including a total of 472 patients with MDD and 680 healthy controls. They could confirm the above-mentioned GMV reductions in patients with MDD in key brain regions implicated in emotion generation and regulation including diffuse bilateral GMV loss in ventrolateral and ventromedial frontal systems extending into the temporal gyri. Moreover, the authors detected GMV reduction in the right parahippocampal and fusiform gyri, hippocampus and bilateral thalamus, parietal lobes and cerebellum in MDD. Schmaal *et al*^10^ performed the largest ever worldwide study by the ENIGMA MDD Working Group on cortical structural alterations in MDD and analysed MRI scans from 2148 patients with MDD and 7957 healthy controls. They also could confirm cortical grey matter loss in the orbitofrontal cortex, anterior and posterior cingulate, insula and temporal lobes. They describe that these effects were more pronounced in the first-episode and adult-onset groups of participants with MDD. Compared with matched controls, the patients with MDD were found to have lower total surface area and regional reductions in frontal regions including the medial orbitofrontal cortex (OFC) and superior frontal gyrus, and primary and higher order visual, somatosensory and motor areas.

Wise *et al*^11^ investigated whether volumetric changes in MDD can be differentiated from those in bipolar disorder. They computed a meta-analysis of VBM studies of 50 data-sets including 1736 individuals with MDD and 2365 healthy controls, and 36 data-sets including 980 individuals with bipolar disorder and 1427 healthy controls. They describe smaller GMV in individuals with MDD in clusters in the dorsomedial and ventromedial prefrontal cortex, including the ACC and bilateral insula in both MDD and bipolar disorder. They also found smaller GMV in the right dorsolateral prefrontal cortex and left hippocampus, along with cerebellar, temporal and parietal regions that were more substantial in MDD when compared with bipolar disorder as well as healthy controls.

Structural connectivity analyses using diffusion tensor imaging (DTI) techniques provides complementary information on these regional reductions in grey matter and allow us to investigate the connectivity between frontal and subcortical structures.^12^ Fractional anisotropy measures how inhomogeneous (anisotropic) the diffusion is in all directions and is commonly used because of its sensitivity to microstructural change, such as altered myelinisation,^13^ although fractional anisotropy is less specific with regard to the type of change.^14,15^ Liao and colleagues^16^ performed an initial meta-analysis of DTI data on a sample of 231 patients with MDD and 261 comparison participants. Despite heterogeneous imaging techniques, decreased fractional anisotropy in the white matter fascicles connecting the prefrontal cortex with cortical (frontal, temporal and occipital lobes) and subcortical areas (amygdala and hippocampus) seems to be a common abnormality in MDD.

Wise *et al*^17^ investigated white matter microstructure differences and similarities between MDD and bipolar disorder by comparing fractional anisotropy between patients and controls. They identified white matter abnormalities in 736 patients with MDD versus 668 controls and 536 patients with bipolar disorder versus 489 controls. They found a significant decrease in fractional anisotropy in the genu of the corpus callosum in both MDD and bipolar disorder and relate these changes to the function of this structure in connecting the two hemispheres of the prefrontal cortex and its role in mood regulation.

All of the above-mentioned studies focused on MDD in general, but relatively little is known about the structural characteristics of TRD. It remains unclear if the underlying brain changes in TRD are simply a more quantitatively severe variant of MDD or whether TRD shows a distinctive pattern of structural brain characteristics, corresponding to different pathophysiology with specific biomarkers and different targets for treatment.

Following the absence of a uniform definition of treatment resistance, the lack of a consistent valid quantification model has been one of the major causes of inconsistency not only affecting the clinical setting but also affecting the research area. Multiple methods for defining and determining treatment resistance have been used ever since the introduction of the concept of TRD in 1974.^18^ At present, the diagnosis of TRD is merely defined by the responsiveness to antidepressants, clinical signs and symptoms. Although the Thase and Rush staging model is commonly used to determine the level of treatment resistance in depression,^19^ it does not integrate potential pathophysiological features of TRD.^20^ In the light of these open questions, we set out to review the current literature on structural brain characteristics of patients with TRD in comparison with both healthy controls and to patients with milder forms of MDD in an attempt to identify potential quantitative and qualitative structural brain abnormalities characterising TRD.

## Method

### Search strategy

**Fig. 1:**
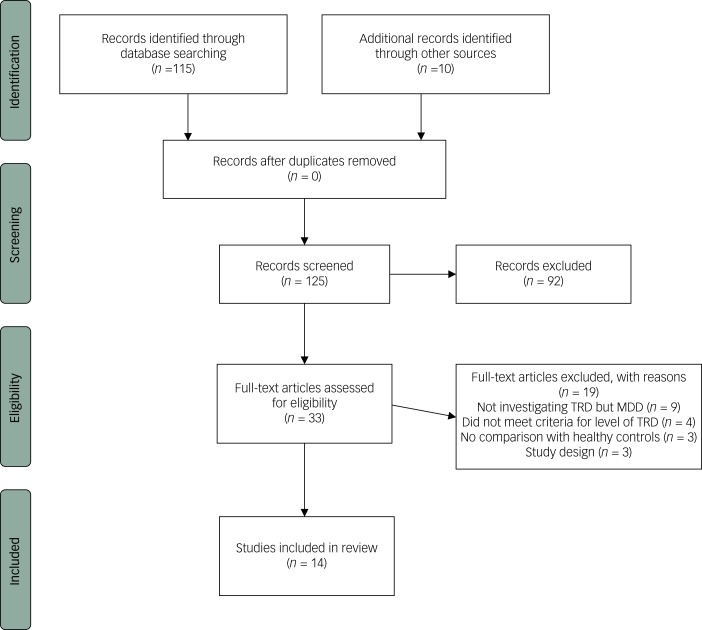
Flow diagram for inclusion in the review.

TRD, treatment-resistant depression; MDD, major depressive disorder.

We performed a literature search in the databases of PubMed and Cochrane Library. Key words used were: “major depressive disorder”, “treatment-resistant”, “treatment-refractory” and “MRI”. This first search yielded 115 articles. Manual search with bibliographic cross-referencing yielded ten more articles. Two authors (M.P.C.K./P.v.E.) reviewed all 125 abstracts. For inclusion in this review we formulated the following criteria: number of patients ≥15; structural brain characteristics investigated using MRI. Structural imaging analyses included ROI analyses or VBM. Moreover, we included DTI or magnetisation transfer imaging (MTI) as other forms of structural imaging. Studies had to include patients with TRD, where TRD was defined by a Thase and Rush stage of ≥II (failure of at least two adequate trials of at least two distinctly different classes of antidepressant medications for at least 6 weeks);^19^ articles that did not use the Thase and Rush model were only included if they met a comparable minimal level of treatment resistance; the investigated patients were at least compared with healthy controls, but ideally to both healthy controls and patients with MDD. In total, 33 articles met inclusion criteria on the basis of the abstract and were subsequently reviewed in more detail by two authors (M.P.C.K./P.v.E.). Out of those, another 19 articles did not meet inclusion criteria and 14 articles were systematically reviewed. For an overview of the selection process see Fig. 1.

### Construction of this review

We will review the results based on MRI methodology (grey or white matter; VBM, MTI, automatic or manual volumetry) and type of analysis (whole brain versus ROI). Results will be weighted as to whether they reflect whole-brain analyses or ROI analyses, which provides results that are not as comprehensive.

## Results

### Characteristics of the studies included in this review

Characteristics of the included studies are listed in Table 1. The median number of patients included in the studies was 24.5 (range 15–115). The mean age of patients included in the studies was 39.9 years (range 26.8–52.1). The male/female ratio of patients included in the studies shows an overall dominance for female patients (8/14 studies) in line with the gender-specific prevalence of MDD. Note that gender was used as covariate in some but not all studies. Twelve studies fulfilled Thase and Rush stage ≥II. Two studies included patients with non-responsiveness to at least two courses of antidepressant medications for at least 4 weeks. The latter did not specify the classes of antidepressant and so did not automatically fulfil Thase and Rush stage II or ≥II.

**Table 1:** Characteristics of included studies

Author (Year)	*n*	Method	Age, years (range or s.d.^a^)	Male/female^b^	Control group (*n*)	Level of therapy resistance
Phillips *et al* (2015)^29^	26	ROI	46 (18–65)	8/18	HC (28)	T&R ≥II
Sun *et al* (2009)^34^	45	ROI	40.8 (18–63)	22/23^b^	HC (30)	T&R ≥II
Furtado *et al* (2008)^33^	45	ROI	37.5 (18–62)	22/23^b^	HC (30)	T&R ≥II
Lan *et al* (2016)^24^	27	WB-VBM	41.5 (16)	9/18^b^	HC (27)	T&R ≥II
Machino *et al* (2014)^26^	29	WB-VBM	39.6 (8.3)	16/13^b^	HC (29)	T&R ≥II
Serra-Blasco *et al* (2013)^23^	22	WB-VBM	49 (8)	4/18	HC/FED/RRD (22/22/22)	T&R ≥III
Zhang *et al* (2009)^27^	15	MTI	33.5 (18–51)	10/5	HC (15)	T&R ≥II
Shah *et al* (2002)^22^	20	WB-VBM	48.9 (9.8)	13/7	HC/RfD (20/20)	T&R ≥III^c^
Ma *et al* (2012)^21^	18	WB-VBM	27.4 (7.74)	11/7	HC/DRT (17/17)	T&R ≥II
Young *et al* (2016)^30^	22	WB-VBM	52.1 (33.9–68)	8/14	HC (21)	T&R ≥IV
Guo *et al* (2012)^20^	23	DTI	27.4 (7.74)	11/12	HC (19)	T&R ≥II
Peng *et al* (2013)^36^	30	DTI	26.9 (5.28)	19/11	HC (25)	T&R ≥III^c^
de Diego-Adelino *et al* (2013)^37^	18	DTI	48.5 (7.3)	3/15	HC/FED/RRD (17/19/15)	T&R ≥II
Maller et *al* (2012 and 2013)^31,32^	115	ROI	39.6 (29–62)	61/54	HC (86)	T&R ≥II

ROI, region of interest; HC, healthy control; T&R, Thase and Rush staging model: ≥II, non-responsiveness to at least two courses of antidepressant medications for at least 6 weeks; ≥III, stage II resistance plus failure of an adequate trial of tricyclic antidepressant; ≥IV, stage III resistance plus failure of an adequate trial of a monoamine oxidase inhibitors; WB-VBM, whole-brain voxel-based morphometry; FED, first-episode depression; RRD, remitted–recurrent depression; MTI, magnetisation transfer imaging; RfD, recovered from depression; DRT, depression responsive to treatment; DTI, diffusion tensor imaging.

a. Range or s.d. depending on the source of information.

b. Gender included as covariate.

c. Non-responsiveness to at least two courses of antidepressant medications for at least 4 weeks.

Four of those 14 studies allowed us to look into dissociating and overlapping structural changes of different stages of major depression by comparing TRD not only to matched healthy controls but also to milder forms of major depression as defined by level of treatment resistance. Two of these four studies compared patients with TRD with healthy controls, patients with first-episode depression and patients with remitted–recurrent depression (see Table 1). One study compared patients with TRD to patients recovered from depression and one study compared patients with TRD with patients with recurrent depression responding to treatment.

### Results of individual studies.

Results of the individual studies have been summarised in supplementary Tables 1 and 2; available online at: https://doi.org/10.1192/bjo.2019.58) whereby we also relate the individual findings to the known meta-analyses of MDD.

### Grey matter studies

#### Whole-brain VBM studies

An important issue to address in this section regarding the individual studies is the issue of statistical inference. Considering the rather small sample sizes of the included studies, the risk of type I errors because of the lack of appropriate control for multiple comparisons is highly likely. Given this, we include the method of correction for multiple comparisons in the separate studies.

##### Studies that included an MDD control group without TRD

Ma *et al*^21^ investigated 18 patients with TRD and compared them with 17 patients with first-episode MDD as well as 17 healthy controls. They used Alphasim (permutation-testint) to correct for multiple comparisons (*P* < 0.005), but applied a looser threshold for the caudate nucleus (*P* < 0.01). Regions with abnormal GMV were also used as seed regions for functional connectivity analyses. In comparison with controls, patients with TRD showed decreased GMV in the right middle temporal cortex and the bilateral caudate, but only the latter was specific for TRD, while volume decreases in the middle temporal cortex were also present in the first-episode MDD group. Moreover, the GMV decrease in the right middle temporal cortex correlated with the duration of illness across all patients with MDD. The decrease in caudate volume in patients with TRD was associated with altered caudate–prefrontal connectivity, which may indicate a substrate for dysregulation of reward mechanisms.

Shah *et al*^22^ compared 20 patients with TRD with 20 patients who were recovered from recurrent MDD (Hamilton Rating Scale for Depression ≤5) and 20 healthy controls. Results were corrected for multiple comparison by means of the cluster-wise correction implemented in SPM. They found reduced grey matter density in the right superior frontal gyrus and the right putamen in the TRD group compared with the combined control and recovered groups. Complementary manual volumetry (not corrected for multiple comparisons) indicated reduced volume of the right prefrontal lobe and the right caudate nucleus (TRD versus non-TRD group, *P* = 0.04; TRD versus control group, *P* = 0.048), which contributed to the pattern of frontostriatal atrophy in TRD that appeared to be more pronounced in more severe illness. In addition, the VBM analysis detected changes in tissue composition in the hippocampus and rostral ACC selectively in the patients with TRD.

Serra-Blasco and colleagues^23^ investigated a group of 22 patients with TRD and compared them with both healthy controls (*n* = 32) and patients with MDD (first-episode depression, *n* = 22 and remitted–recurrent depression, *n* = 22). Results were familywise-error (FWE)-corrected at *P* < 0.05 in SPM. Compared with healthy controls, the patients with TRD showed a broad set of clusters of reduced GMV in the anterior cingulate gyrus, superior, medial and inferior frontal gyri, the insula, the parahippocampal gyrus and the transverse temporal gyrus (FWE-corrected at *P* < 0.05; threshold: 100 voxels). Except for smaller GMV in the medial frontal gyrus, the patients with TRD revealed qualitative GMV changes compared with the first-episode group in the precentral gyrus, the medial frontal gyrus, the insula, the transverse temporal gyrus, the inferior parietal lobule and the posterior cingulate. In subsequent analyses using the automated segmentation program FreeSurfer the TRD group revealed smaller volumes of the right medial frontal gyrus and the left insula in comparison with those in the first-episode group. Again, these changes correlated with illness duration.

##### Studies that only included a healthy control group

Lan *et al*^24^ investigated 27 patients with TRD with a current major depressive episode and compared them with 27 healthy controls. Results were FWE-corrected (*P* < 0.05, cluster threshold 500 voxels) in SPM. They found six clusters of decreased GMV in the TRD group (FWE-corrected at *P* < 0.05; threshold: 500 voxels). These clusters were located in the left medial prefrontal cortex, including the ACC, the left lingual gyrus, the left middle and superior temporal gyrus, the left insula, the posterior lobe of the right cerebellum and the right angular gyrus. Additionally, the TRD group were treated with high-frequent repetitive transcranial magnetic stimulation over the left dorsolateral prefrontal cortex over 5 weeks, which resulted in a response in 33% of the patients. Post-treatment imaging showed that four of the clusters (the left ACC, the left insula, the left superior temporal gyrus and the right angular gyrus) showed selective GMV increase after transcranial magnetic stimulation treatment. In addition, grey matter increase of the ACC correlated with clinical improvement. These findings are in line with state-related neuroplasticity changes that are also reported in non-TRD forms of depression.^25^

Machino *et al*^26^ investigated 29 patients with TRD in comparison with healthy controls and found significantly smaller GMV in ventral and dorsal parts of the ACC, the cerebellum and in the right superior frontal gyrus. These results were not corrected for multiple comparison (statistical threshold: *P* uncorrected < 0.001; cluster: 50 voxels). Moreover, Lan *et al*^24^and Machino *et al*^26^ described reduced GMV in the right cerebellum in TRD compared with healthy controls.

In summary, VBM studies reveal quantitative differences between patients with TRD and with milder forms of MDD and HCs in various fronto-temporolimbic regions as summarised in supplementary Tables 1 and 2. A selective structural deficit in TRD compared with other forms of MDD has been found in the superior frontal gyrus, the middle and inferior temporal gyrus and the caudate nucleus.

#### MTI

Zhang *et al*^27^ investigated 15 patients with clinically defined TRD and 15 matched healthy controls combining MTI and standard *T*_1_-weighted imaging for subsequent VBM analyses. MTI is a MRI technique based on the selective saturation of protons bound to macromolecules such as myelin thereby identifying damage to myelin and to other cellular structures, such as the axonal membranes.^28^ Surprisingly and possibly related to the low number of participants, VBM revealed no morphological abnormalities in the TRD group compared with the control group. However, a reduced magnetisation transfer ratio was observed in the ACC, insula, caudate tail and amygdala–parahippocampal areas.

#### ROI studies

The structures involved in TRD according to the ROI studies reviewed are the amygdala, the hippocampus, the ACC, the entorhinal cortex and the corpus callosum. Phillips *et al*^29^ investigated potential differences during an approximate 1-year follow-up period in 26 patients with TRD compared with 28 matched healthy controls in six ROIs (hippocampus, rostral middle frontal gyrus, orbitofrontal cortex, rostral and caudal anterior cingulate cortices and inferior temporal gyrus). While surprisingly there were no differences at baseline, after 1-year the non-remitting group showed decreasing volume and cortical thinning over time in the rostral middle frontal gyrus, orbitofrontal cortex and inferior temporal gyrus, whereas the remitted group showed increasing volume of thickness over follow-up in these regions.

Young *et al*^30^ combined grey and white ROI investigations in a group of 22 patients with TRD and 21 healthy controls by combining VBM and tract based spatial statistics (TBSS) DTI. They focused on frontolimbic structures and in particular targeted the amygdala, hippocampus and ACC and connecting tracts. The VBM analysis indicated that patients with TRD showed reduced GMVs relative to healthy controls in the left medial OFC and bilateral hippocampus with a particularly prominent cluster in the left hippocampus. TBSS revealed elevated fractional anisotropy values in the left angular bundle and right uncinate fasciculus.

Using manual segmentation of the hippocampus, Maller *et al*^31,32^ investigated 115 patients with different stages of MDD and concluded that particularly the tail section of the hippocampus showed reduced volume in patients with TRD. Moreover, patients with TRD had significantly more sulcal cavities and their presence and length was associated with ageing.

As an important structure of the hippocampal formation Furtado *et al*^33^ investigated the entorhinal cortex in 45 patients with TRD and 30 healthy age- and gender-matched controls. They found significant reductions in the left entorhinal cortex only in female patients.

Sun *et al*^34^ investigated the morphology of subregions of the corpus callosum in 45 patients with TRD compared with 30 healthy controls. Previous literature found larger total corpus callosum and larger anterior and posterior quarters of the corpus callosum in MDD (not specified as TRD).^35^ However, Sun and colleagues^34^ could not replicate those findings and only found reduced volume of one part of the corpus callosum in the TRD group.

### White matter: DTI studies (whole brain)

Guo *et al*^20^ investigated the white matter integrity in TRD and found three brain white matter tracts with lower fractional anisotropy compared with healthy controls. These tracts include the right anterior limb of the internal capsule, the body of the corpus callosum and bilateral external capsule. Despite the same methodology Peng *et al*^36^ could not replicate the findings of Guo *et al*^20^ but found reduced fractional anisotropy in the left limbic lobe uncus, left middle frontal gyrus and right cerebellum posterior lobe in patients with TRD compared with healthy controls.

Additionally, de Diego-Adelino *et al*^37^ found decreased fractional anisotropy in patients with TRD compared with both healthy controls and patients with first-episode depression. Whole-brain analysis revealed a generalised significant reduction in fractional anisotropy in TRD compared with healthy controls mostly affecting bilateral inferior fronto-occipital fasciculus, bilateral inferior longitudinal fasciculus, bilateral superior longitudinal fasciculus, forceps major and forceps minor, the body of the corpus callosum and bilateral cingulum. A significant decrease in fractional anisotropy was also observed in patients with TRD compared with patients with a first episode affecting the body of the corpus callosum, bilateral superior longitudinal fasciculus, forceps minor, forceps major, bilateral cingulum and bilateral inferior longitudinal fasciculus.

## Discussion

To the best of our knowledge this is the first review of structural brain changes in TRD both in comparison with healthy controls and with non-TRD groups with MDD. Considering the results, some methodological limitations have to be taken into account. Sample sizes of the included studies were often small, limiting statistical inference of the results. As a consequence, described structural differences in TRD may be the result of noise instead of real differences. In most studies patients with TRD were only compared with healthy controls but no other MDD groups (first-episode, remitted–recurrent depression). This latter issue is crucial in the differentiation of TRD from MDD to understand whether TRD represents a continuum from less severe forms of MDD or possibly comes with its own structural brain characteristics. Although the comparison between patients with TRD and non-TRD groups of MDD should address the aforementioned issues, we cannot rule out that the patients with TRD were also characterised by longer duration of depression and possibly affected by a larger allostatic load and larger exposure to medication with possible neurobiological effects. There was only one study that correlated measures of treatment resistance (medication load) and duration with structural changes.^23^

Below we will discuss the relevant findings per region. For the purpose of this review we regard the studies that compared TRD with a non-TRD control group as having the highest level of evidence. Moreover, we focus our interpretation of results on converging evidence from separate studies that identified the same structures or converging evidence from different MRI modalities.

### Converging evidence

From the studies that used a MDD control group, two out of three studies identified decreased volume of the caudate nucleus.^21,22^ Based on these results the pattern of frontostriatal atrophy seems to be the most discriminative aspect of TRD versus milder forms of MDD. Hence, Wise *et al*,^11^ describe decreased GMV in the caudate nucleus in MDD when compared with healthy controls as well. On the one hand, this finding may illustrate the fact that GMV loss in the caudate nucleus is not discriminating TRD from MDD as it is seen in both groups. On the other hand, these findings may illustrate a continuum of disease-related structural changes starting at MDD and progressing to TRD as the GMV loss is already present in MDD but is significantly greater in TRD when compared with MDD, and hence could present a progression of disease.

A decrease in insula volume was only found by Serra-Blasco *et al*^23^ in comparison with a MDD group but it was also found in comparison with healthy controls, so it may not be specifically related to TRD.^24^ The level of treatment resistance was included in this study in which they describe a negative correlated decrease in the right medial frontal cortex and left insula. GMV loss in the insula was also described in multiple meta-analyses investigating MDD versus healthy controls,^9–11^ which again could reflect a progression of disease from MDD to TRD but does not support this structure as being discriminative for TRD. Moreover, decreased structural integrity of the corpus callosum was implicated in three studies with different modalities,^20,27,37^ one of which included a non-TRD control group.^37^ Wise *et al*,^17^ describe decreased fractional anisotropy in the corpus callosum in MDD compared with both healthy controls and patients with bipolar disorder. Whereas Wise *et al*,^17^ do not include level of treatment resistance in their analysis it remains unclear whether the investigated group of patients with MDD in their analysis perhaps also includes patients who were treatment resistant as well, thereby possibly confounding results with respect to our comparison.

In multiple studies that compared patients with TRD directly with healthy controls, the ACC showed decreased GMV^24,26,27^ next to the cerebellum.^24,26,36^ These results are possibly not discriminative for TRD as these findings were not present in the studies that compared patients with TRD- with a non-TRD MDD group. Supporting evidence against the discriminative properties of the ACC as well as the cerebellum for TRD are given by the meta-analyses of Arnone *et al*,^9^ Wise *et al*^11^ and Schmaal *et al*,^10^ which describe these alterations in MDD as well. On the other hand, surprisingly the basal ganglia volume loss^21,22^ was not found when comparing TRD and healthy control groups, except for the study by Zhang *et al*^27^ that found reduced MTR in the tail of the caudate nucleus. This study also included duration of illness.

Two out of three ROI studies found reduced hippocampal volume^30–32^ while most whole-brain studies failed to identify differences in hippocampal volume. In the remains of this discussion we will take a closer look at the above-mentioned structures in order of level of evidence from our review.

### Basal ganglia

Decreased volume of the caudate nucleus seems to be specific to TRD.^10,21^ Additional connectivity analysis of functional MRI data by Ma *et al*,^21^ showed that this decrease in caudate nucleus volume is accompanied by aberrant functional connectivity, both increased and decreased, to frontal regions, which in turn often show structural changes mainly as a function of illness severity. This may support the hypothesis that TRD is characterised by frontostriatal atrophy, which could be a neural correlate of deficient reward mechanisms in TRD on top of other neurocognitive mechanisms maintaining MDD. More supporting evidence comes from the fact that the GMV of the putamen is not found to be significantly decreased in MDD and thus the putamen may be a specific structure in the differentiation of TRD from MDD.

### Medial prefrontal cortex

The medial prefrontal cortex is an important monitoring hub of the prefrontal cortex that supports reward, decision-making and memory functions.^38^ Given its broad cognitive functions in the context of processing of emotional information, it is not surprising that the medial prefrontal cortex plays a crucial role in the pathogenesis of depression. Indeed our review supports the important role of this region since volume decreases were found in two out of three studies^23,24^ and were also apparent in the direct comparison between TRD and first-episode depression.^23^ It may be relevant to adjust the current neuropsychological testing of patients with MDD such that medial prefrontal cortex function is more specifically tested and could possibly be used for further diagnostics as well as becoming more of a focus for treatment.

### Medial and superior frontal gyrus

A decrease in volume of the medial and superior frontal gyri was found in studies that used a non-TRD MDD group as well as studies comparing TRD with healthy controls only.^22,23,26^ Although hypoactivity in these executive control regions is a common finding in both MDD and TRD,^9–11^ Serra-Blasco *et al*^23^ showed a negative correlation between the volume of the medial frontal gyrus and duration of illness. The evidence included in this review does not give enough support to differentiate between effect of chronicity over time and TRD-specific structural alterations of these frontal regions.

### Insula

In line with the aforementioned quantitative changes, smaller insula volumes were found in the participants with TRD compared with the first-episode MDD group^23^ and healthy controls^24^ that was negatively correlated with duration of illness. The insula is involved in the monitoring of internal states, and emotional and sensorimotor processing, which is negatively affected in MDD.^39^ Interestingly, the insula is implicated by several studies as a correlate of treatment response.^40^ Even in severe forms of TRD treated with electroconvulsive therapy (ECT) increases in cortical thickness of the insula was specific to responders.^39^

### Corpus callosum

The role of the corpus callosum in MDD and TRD in particular remains elusive since only ROI studies found decreased white matter integrity in patients with TRD.^20,27,37^ Whereas Wu *et al*^35^ described larger total corpus callosum and larger anterior and posterior quarters of the corpus callosum in MDD, Sun *et al*^34^ on the contrary found reduced volume of the corpus callosum in one subregion in patients with TRD. Future studies should investigate changes in the corpus callosum of patients with TRD compared with other patients with MDD in whole-brain analyses taking larger sample sizes also into account.

### ACC

Our review once more supports the strong involvement of the ACC in TRD and MDD in general as this structure by far reveals the most consistent significant reduction in volume in TRD across multiple studies (see supplementary Tables 1 and 2) in comparison with healthy controls. Nevertheless, this structure is not specifically affected in TRD as is shown by the loss of volume in non-TRD groups of patients with MDD in the TRD studies as well as meta-analyses investigating structural characteristics in MDD.^9–11^ Our review supports the hypothesis that volume reduction of the ACC already exists in the early course of depression^8,23^ and thus may represent a stage-independent trait marker of the disease. Since structural differences have even been found in vulnerable individuals volume reduction in ACC^41^ may even serve as a structural endophenotype of depression but does not specifically characterise TRD. Early structural changes to the ACC could indeed account for aberrant functional connectivity of limbic–cortical pathways, which has been proposed in several pathophysiological models of depression.^42^ Normal functioning of the rostral anterior cingulate, with its direct connections to dorsal and ventral areas, is hence required for reciprocal compensatory changes in depression.

### Cerebellum

Where Lan *et al*^24^ and Machino *et al*^26^ not only agree on reduced GMV in the ACC in TRD, they both show volume reduction of the cerebellum, which was not, however, supported by Serra-Blasco *et al*.^23^ However, loss of GMV of the cerebellum does not seem to be specifically related to TRD as it is described in MDD studies too.^9,11^ Transdiagnostic research has implied cerebellar involvement in emotion regulation,^43^ and in line with this, reduced GMV is common in attention-deficit hyperactivity disorder as well. Cerebellar involvement in TRD does seem plausible, but with affirmative literature still missing.

### Hippocampus

Only the meta-analyses found smaller hippocampal volume among patients with MDD^7,44^ suggesting that if there are TRD-specific changes these are subtle and current studies are underpowered to detect them. Shah *et al*^22^ suggest that a change in tissue composition rather than volume characterises TRD in the hippocampus. Young *et al*,^30^ showed that the hippocampus is altered by TRD only in comparison with healthy controls but strengthen their results by combining the VBM method with a TBSS of the white matter, which revealed elevated fractional anisotropy values in the related fibre tracts of the hippocampus (angular bundle and unicated fasciculus).

Maller *et al*^31,32^ hypothesise that the hippocampus tail may be more sensitive to the pathophysiology of depression than other parts of the hippocampus. They explain that in terms of structural connections the posterior hippocampus projects to the dorsolateral region of the prefrontal cortex, which is a well-known important structure in TRD. Myelinated fibres enter and leave the posterior hippocampus along the tail, which contains relatively few neuronal elements compared with the body segment. The loss of GMV overall could be too small to reach a level of significance and so would not be detected in the standard measurements. It must be noted that whole-brain analysis is a more comprehensive way to analyse differences, but it needs much higher effect sizes (given the same sample size) to yield significant results, because of multiple comparison.

All together, even though the evidence given by the literature included in this review about the involvement of the hippocampus in TRD is not congruent, the hippocampus remains an important structure for future research into TRD given also its potential role in treatment effects of ECT in TRD.^45^

### Superior longitudinal fasciculus

The superior longitudinal fasciculus is a big bundle of association fibres that connects the dorsolateral prefrontal cortex and other frontal regions with the temporal, parietal and occipital lobes. The cingulum bundle lies within the cingulated gyrus and is an important association pathway linking prefrontal and parahippocampal cortices. The inferior longitudinal fasciculus is an association fibre tract that connects the occipital and temporal lobes, including the hippocampus and amygdala.^46^ Whereas the superior longitudinal fasciculus as well as the inferior longitudinal fasciculus are found to have altered integrity in TRD by de Diego-Adelino *et al*,^37^ these structures are not described by the meta-analyses investigating MDD^9–11^ and thus may be an interesting structure for future investigation of structural differences between MDD and TRD.

### Implications and limitations

The main finding of this study is without a doubt the lack of convergence. Even though occasional overlap is found there are no evident converging structural changes in TRD that are replicated by different research groups. Hypothetically this could be the result of the small sample size of the individual studies leading to type I errors. Given the need to learn more about the neural underpinnings of TRD in comparison with MDD, it is important that large consortia like ENIGMA focus on an investigation of these aspects.

An important limitation of this field of research is the lack of agreement about the defining of patients with TRD. Criteria for therapy resistance become more and more clarified these days. An important issue to realise is the fact that the group of patients which was used as a reference for MDD was determined before TRD was considered to be a different group compared to MDD. Most likely, the group of patients with MDD, as used in the MDD studies, contained patients with TRD as well, and so this could have biased the outcome of studies comparing these results with patients with TRD.

Other potential confounders associated with TRD that have to be taken into account are illness duration, age and history of medication use. Individuals with longer duration of illness could potentially be more likely to run a treatment-resistant course. On the other hand, one could hypothesise that if the duration of disease continues, eventually every individual could potentially become treatment resistant. The effects of age on the level of treatment resistance would be very interesting to investigate as well as the history of medication use. One could hypothesise that older age and use of more medication could result in a more vulnerable brain and so could influence the course of depression towards a level of treatment resistance. To date, no relevant data about these confounders are included in the literature and so could not be corrected for. The fact that the reviewed articles did not control for severity of depression in our opinion is a relevant issue to address. Given the small sample size of the individual studies, one would have to pool data for correction of severity, which might be an interesting question for a future study.

Another difficulty in comparing results of research in this area is the fact that multiple analysing techniques are used to map differences in brain structure during the course of depression. Most important, the currently available data cannot sufficiently distinguish between a ‘shared continuum hypothesis’ and a ‘different entity hypothesis’. Our review clearly suggests that although there is some overlap in affected brain regions between milder forms of MDD and TRD, TRD also comes with specific alterations mainly in the putamen, inferior frontal gyrus, precentral gyrus, angular- and post-central gyri together with specific mainly parietal white matter tract changes.

The most legitimate way to investigate structural characteristics in TRD would be to compare a group of patients with TRD with patients with MDD who do not fulfil criteria for TRD, as well as healthy controls. Moreover, to make a clear subdivision between the different types of major depression, future research would ideally want to including subgroups of patients with MDD with first-episode depression versus remitted–recurrent depression and compare both groups with a group with TRD; the putamen, inferior frontal gyrus, precentral gyrus, angular- and post-central gyri together with specific white matter tracts clearly serve as important candidate regions.

## Supplementary material

For supplementary material accompanying this paper visit http://dx.doi.org/10.1192/bjo.2019.58. click here to view supplementary material
