# Supplementary material for: Structural brain characteristics in treatment-resistant depression: review of magnetic resonance imaging studies
Source: BJPsych Open. 2019 Sep 2;5(5):e76. doi: 10.1192/bjo.2019.58 (PMC6737518; doi:10.1192/bjo.2019.58)
Supplement: Supplementary file 1 [file S2056472419000589sup001.docx]

Supplementary Table 1 Results of included studies: significant gray matter volume reductions; TRD compared to different control groups and compared to known characteristics in MDD

|  | | | **Frontal** | | | | | | | | | **Parietal** | | **MTL** | | | | **Temporal** | | | | | | **Cerebellum** | **Striatum** | |
| --- | --- | --- | --- | --- | --- | --- | --- | --- | --- | --- | --- | --- | --- | --- | --- | --- | --- | --- | --- | --- | --- | --- | --- | --- | --- | --- |
|  |  |  | Frontal inferior opercularis | Orbitofrontal gyrus | Rostral middle frontal gyrus | Precentral gyrus | Anterior cingulate cortex | Frontal gyrus | | | Cingulated gyrus | Post central gyrus | Angular gyrus | Insula | Enthorhinal cortex | Hippocampus | Parahippocampal gyrus | Transversotemporal gyrus | Temporal gyrus | | | Lingual gyrus | Fusiform gyrus |  | Putamen | Caudate nucleus |
|  |  |  |  |  |  |  |  | medial | superior | inferior |  |  |  |  |  |  |  |  | medial | superior | inferior |  |  |  |  |  |
| *Whole brain VBM studies* |  | **Control group** |  |  |  |  |  |  |  |  |  |  |  |  |  |  |  |  |  |  |  |  |  |  |  |  |
|  | **Serra-Blasco** | HC |  |  |  |  | x | x | x | x | x | x |  | x |  |  | x | x |  |  | x^2^ |  |  |  |  |  |
|  |  | 1E |  |  |  | x |  | x |  |  |  |  |  | x |  |  |  | x |  |  |  |  |  |  |  |  |
|  |  | RRD |  |  |  |  |  |  |  |  |  |  |  |  |  |  |  |  |  |  |  |  |  |  |  |  |
|  | **Shah** | HC |  |  |  |  |  |  | x |  |  |  |  |  |  |  |  |  |  |  |  |  |  |  | x | x |
|  |  | RD |  |  |  |  |  |  | x |  |  |  |  |  |  |  |  |  |  |  |  |  |  |  | x | x |
|  | **Ma** | HC |  |  |  |  |  |  |  |  |  |  |  |  |  |  |  |  | x |  |  |  |  |  |  | x |
|  |  | 1E |  |  |  |  |  |  |  |  |  |  |  |  |  |  |  |  | x |  |  |  |  |  |  |  |
|  | **Machino** | HC |  |  |  |  | x |  | x |  |  |  |  |  |  |  |  |  |  |  |  |  |  | x |  |  |
|  | **Lan** | HC | x |  |  | x | x | x |  |  |  |  | x | x |  |  |  |  | x | x |  | x | x | x |  |  |
| *MTI study* | **Zhang** | HC |  |  |  |  | x |  |  |  |  |  |  |  |  |  |  |  |  |  |  |  |  |  |  | x |
| *Region of interest studies* | **Furtado** | HC |  |  |  |  |  |  |  |  |  |  |  |  | x |  |  |  |  |  |  |  |  |  |  |  |
|  | **Maller** | HC |  |  |  |  |  |  |  |  |  |  |  |  |  | x |  |  |  |  |  |  |  |  |  |  |
|  | **Philips** | HC |  | x | x |  |  |  |  |  |  |  |  |  |  |  |  |  |  |  |  |  |  |  |  |  |
|  | **Young** | HC |  |  |  |  |  | x |  |  |  |  |  |  |  | x |  |  |  |  |  |  |  |  |  |  |
| *Meta-analyses MDD* | **Wise (2017)** | HC |  |  |  |  | x | x | x |  |  |  |  | x |  |  | x |  |  | x |  |  | x |  |  | x |
|  |  | BD |  |  |  |  |  | x |  |  |  |  |  |  |  | x | x |  |  |  | x |  | x | x |  |  |
|  | **Arnone** | HC |  |  |  |  | x | x | x |  | x |  |  | x |  | x | x |  |  | x | x | x | x | x |  |  |
|  | **Schmaal** | HC |  |  |  |  | x |  | x |  |  |  |  | x |  |  | x |  | x |  | x |  | x | x |  |  |

MTL=mediotemporal lobe, BD= bipolar disorder, ^2^= longitudinal analysis. HC= healthy controls; RD=recovered from depression; 1E= 1^st^ episode depression;
 RRD= remitted-recurrent depression, = highest level of evidence (comparison multiple groups), = only comparison to HC, = GM volume reductions in MDD.

Supplementary Table 2 Results of included DTI/MTI studies: white matter tracts with lower FA; TRD compared to different control groups and compared to known characteristics in MDD

|  | | | Frontal | | Parietal | | | | MTL | | | | Temporal | Cerebellum |
| --- | --- | --- | --- | --- | --- | --- | --- | --- | --- | --- | --- | --- | --- | --- |
|  |  |  | Anterior cingulate cortex | Inferior fronto-occipital fasciculus | Longitudinal fasciculus | | Forceps major | Forceps minor | Corpus callosum | Internal capsule | Uncus | Cingulum | External capsule |  |
|  |  |  |  |  | Inferior | Superior |  |  |  |  |  |  |  |  |
| *DTI*  *studies* |  | Control group |  |  |  |  |  |  |  |  |  |  |  |  |
|  | **Guo** | HC |  |  |  |  |  |  | X | X |  |  | X |  |
|  | **Peng** | HC |  |  |  |  |  |  |  |  | X |  |  | X |
|  | **Diego-Adelino** | HC |  | X | X | X | X | X | X |  |  | X |  |  |
|  |  | 1E |  |  | X | X | X | X | X |  |  | X |  |  |
| *Region of interest study* | **Sun** | HC |  |  |  |  |  |  | X |  |  |  |  |  |
| *MTI study* | **Zhang** | HC | X |  |  |  |  |  | X |  |  |  |  |  |
| *Meta-analysis MDD* | **Wise (2016)** | HC |  |  |  |  |  |  | X |  |  |  |  |  |
|  |  | BD |  |  |  |  |  |  | X |  |  |  |  |  |

DTI = diffusion tensor imaging, MTI = Magnetization transfer imaging, * = compared to healthy controls, ** = compared to healthy controls, first episode depression and recurrent/remitted depression
